# Performance of Positron Emission Tomography-Computed Tomography and Bone Marrow Biopsy in Detecting Bone Marrow Infiltration in Lymphoma Cases

**DOI:** 10.4274/tjh.galenos.2020.2019.0361

**Published:** 2020-11-19

**Authors:** Mahmut Büyükşimşek, İrem Kolsuz, Abdullah Evren Yetişir, Mert Tohumcuoğlu, Ali Oğul, Cem Mirili, Semra Paydaş, İsa Burak Güney

**Affiliations:** 1 Çukurova University Faculty of Medicine, Department of Medical Oncology, Adana, Turkey; 2 Çukurova University Faculty of Medicine, Department of Internal Medicine, Adana, Turkey; 3 Adana Health Practice and Research Center, Department of Medical Oncology, Adana, Turkey; 4 Atatürk University Faculty of Medicine, Department of Medical Oncology, Erzurum, Turkey; 5 Çukurova University Faculty of Medicine, Department of Nuclear Medicine, Adana, Turkey

**Keywords:** Lymphoma, Bone marrow infiltration, PET/CT, Bone marrow biopsy

## Abstract

**Objective:**

Bone marrow infiltration (BMI) affects the stage diagnosis, and treatment of lymphoma. We aimed to evaluate the performance of bone marrow biopsy (BMB) and positron emission tomography-computed tomography (PET/CT) in detecting BMI in lymphoma patients.

**Materials and Methods:**

A total of 269 non-Hodgkin’s lymphoma (NHL) and 110 Hodgkin’s lymphoma (HL) patients were evaluated retrospectively. Sensitivity, negative predictive value (NPV), and accuracy were calculated for PET/CT and BMB in detecting BMI.

**Results:**

Sensitivity, NPV, and accuracy for PET/CT in detecting BMI in NHL cases were 65%, 78%, and 84.4%, respectively, while they were 55%, 73.4%, and 79.9% for BMB. PET/CT performance for diffuse large B-cell lymphoma and follicular lymphoma was better than that of BMB, whereas the performance of BMB was better for mantle-cell lymphoma, Burkitt’s lymphoma, and primary mediastinal B-cell lymphoma. Sensitivity, NPV, and accuracy for PET/CT in HL cases were 91.3%, 97.75%, and 98.18%, respectively, while they were 56.52%, 89.69%, and 90.91% for BMB. Due to BMB, 43 (15.9%) patients in the NHL group and 2 (1.8%) patients in the HL group were protected from downstaging.

**Conclusion:**

Although their results vary according to NHL subtypes, PET/CT and BMB are complementary methods in determining BMI. In HL, PET/CT is an important diagnostic tool for detecting BMI, and BMB is not necessary in a significant proportion of cases.

## Abstract (tr)

**Amaç:**

Kemik iliği infiltrasyonu (Kİİ), lenfoma evresini, sağkalımı ve tedaviyi etkiler. Lenfoma hastalarında Kİİ’yi saptamada kemik iliği biyopsisi (KİB) ve pozitron emisyon tomografi-bilgisayarlı tomografinin (PET/BT) performanslarını değerlendirmek istedik.

**Gereç ve Yöntemler:**

İki yüz altmış dokuz non-Hodgkin lenfoma (NHL) ve 110 hodgkin lenfoma (HL) hastası retrospektif olarak değerlendirildi. Kİİ’yi saptamada PET/BT ve KİB için duyarlılık, negatif prediktif değer (NPD) ve doğruluk hesaplandı.

**Bulgular:**

NHL olgularında Kİİ’yi saptamada PET/BT için duyarlılık, NPD ve doğruluk sırasıyla %65, %78 ve %84,4 iken KİB için %55, %73,4 ve %79,9 idi. Diffüz büyük B-hücreli lenfoma ve folliküler lenfoma için PET/BT performansı KİB’den daha iyi iken; mantle-cell lenfoma, Burkitt’s lenfoma ve primer mediastinal B-hücreli lenfomada KİB performansı daha iyiydi. HL olgularında PET/BT için duyarlılık, NPD ve doğruluk sırasıyla %91,3, %97,75 ve %98,18 iken KİB için %56,52, %89,69 ve %90,91 idi. KİB yapılması ile NHL grubunda 43 (%15,9), HL grubunda ise 2 (%1,8) olgunun düşük evrelenmesinden korunmuş olundu.

**Sonuç:**

Kİİ’yi saptamada NHL alt tiplerine göre değişmekle birlikte PET/BT ve KİB birbirini tamamlayıcı yöntemlerdir. HL’de ise Kİİ’yi saptamak için PET/BT önemli bir tanı aracıdır ve KİB olguların önemli kısmında gerekli değildir.

## Introduction

Lymphoma prognosis and treatment largely depend on the stage at the time of diagnosis [1,2]. Bone marrow infiltration (BMI) is critical in lymphoma staging, treatment selection, and prognosis as it is considered a stage 4 disease [3]. Bone marrow biopsy (BMB) has been considered the gold standard for detecting BMI until recently, despite the obvious disadvantages of being an invasive method, being painful, and causing procedural anxiety, bleeding, and fractures at the biopsy site [4,5]. 18-F fluorodeoxyglucose (FDG) positron emission tomography with computed tomography (PET/CT) is a non-invasive method for detecting extramedullary disease and evaluating whole-body bone marrow. These advantages have been suggested to make PET/CT the gold-standard method for staging lymphoma and evaluating response to treatment, as well as for PET/CT to replace BMB for bone marrow evaluation [6]. Although the ability of PET/CT to evaluate BMI has been investigated in several studies recently, it is still a matter of debate whether it can replace BMB [7,8,9,10]. In this study, we aimed to compare the diagnostic value of PET/CT and BMB in the detection of BMI in newly diagnosed non-Hodgkin’s lymphoma (NHL) and 110 Hodgkin’s lymphoma (HL) patients.

## Materials and Methods

### Study Design and Patients

This was a single-center retrospective study. In 2011-2018, in the Medical Oncology Department of Çukurova University, patients who were diagnosed with HL or NHL over the age of 18 and who underwent BMB and PET/CT as a part of pretreatment staging were included in the study. The time interval between the two procedures was a maximum of 2 weeks, and the patients did not use corticosteroid or chemotherapy before both procedures and they did not have any other malignancies. Infection, anemia, and other pathological conditions that may cause false-positive results on PET/CT imaging were excluded by performing complete blood counts, biochemical tests, cultures, and physical examinations. In addition, patients who underwent blood transfusion prior to PET/CT were excluded. Medical records of 401 patients were reviewed and the subgroups of low-grade NHL, chronic lymphocytic leukemia/small lymphocytic lymphoma, and nodular lymphocyte-predominant HL were excluded. For the NHL group, diffuse large B-cell lymphoma (DLBCL), grade 3 follicular lymphoma, mantle cell lymphoma, Burkitt’s lymphoma, and primary mediastinal large B-cell lymphoma (PMBCL) subtypes were included in the study. Results of 269 NHL and 110 HL patients were evaluated. The patients were staged according to the Ann Arbor staging system.

### Bone Marrow Biopsy

All BMB and aspiration samples were taken unilaterally from the dorsal iliac crest, 10-15 mm in size and untargeted, and routinely fixed in formol and embedded in paraffin. They were then stained with hematoxylin-eosin and Giemsa and morphologically evaluated by experienced hematopathologists in our center. As a rule, all samples were stained with CD19 and CD20 for B cell origin, CD3 and CD5 for T-cell origin, and CD15 and CD30.

### 18F-FDG PET/CT

Patients fasted for at least 6 h and serum glucose levels were below 120 mg/dL in all patients. 18F-FDG PET/CT scans were acquired 60-90 min after intravenous administration of 18F-FDG (350-370 MBq) and performed as whole-body scans (from the base of the skull to mid-thigh). 18F-FDG PET/CT images were obtained using combined PET/CT Siemens equipment (Biograph 6, Siemens Medical Systems USA). Both the PET and CT scans were obtained during normal tidal breathing. The total acquisition time varied between 25 min and 35 min per patient. PET images were reconstructed with CT-derived attenuation correction using the ordered subset expectation maximization algorithm. The attenuation-corrected PET images, CT images, and fused PET/CT images displayed as coronal, sagittal, and transaxial slices were viewed on a Symbia workstation (Siemens Healthcare). All PET/CT images were assessed for BMI by experienced nuclear medicine physicians without the results of the BMBs. Most of the image interpretation was performed through qualitative (visual) analysis, considering the presence or absence of BMI using glucose activity in the liver as a reference. PET/CT is highly sensitive in demonstrating bone marrow focal involvement in patients with aggressive NHL and HL. As previously described, PET/CT was considered positive when there was focal or multifocal bone marrow or bone involvement [11]. Diffuse BMI in PET/CT was not considered positive because it may be due to inflammation or benign causes [12,13].

### Statistical Analysis

The diagnosis of BMI by lymphoma was made by either positive BMB or positive PET/CT [14]. Accuracy was calculated according to the formula (TP+TN)/(TP+TN+FP+FN), while sensitivity was calculated according to the formula (TP/TP+FN). The negative predictive value (NPV) was calculated using (TN/TN+FN). Here, TP denotes true positive cases, TN true negative cases, FP false positive cases, and FN false negative cases. Since BMB or PET/CT positivity indicates BMI, the positivity of the test performed for it was considered as true positive. Patients with both tests negative were considered as true negative. If one test was positive and the other negative, it was considered as false negative. Since all positive results were considered significant for that test, no false positive results were accepted. Median and range values for continuous variables and percentages for categorical variables are reported. Differences between groups were tested by the chi-square method. Nominal values were compared using kappa statistics, and p<0.05 was considered statistically significant. Statistical analysis was done with SPSS software.

## Results

A total of 269 patients with NHL and 110 patients with HL were included in the study. Their clinical and demographic features are shown in Table 1.

### Group I - NHL

The median age of NHL patients at diagnosis was 52 years (range: 18-80). In this group, 159 patients were male and 110 patients were female. Sixty-six of the patients (24.5%) had BMI in the BMB, and 78 (28.9%) had BMI according to PET/CT findings. Thirty-eight of 78 patients had focal involvement, while 40 patients had multifocal involvement. Focal involvement was observed in 10 of 24 patients who were found to be BMI-positive according to both BMB and PET/CT findings, and multifocal patterns were observed in 14 of these cases.

### Impact of PET/CT and BMB Findings on Staging

According to PET/CT findings, 45 patients were evaluated as stage 1, 58 patients as stage 2, 101 patients as stage 3, and 65 patients as stage 4. Six patients with stage 1, 7 patients with stage 2, and 30 patients with stage 3 were evaluated as stage 4 because of having positive BMB. Due to the BMB, 42 patients (15.6%) were upstaged compared to the PET study. Of these patients, 28 had DLBCL, 4 had follicular lymphoma, 8 had mantle cell lymphoma, 1 had Burkitt’s lymphoma, and 1 had PMBCL subtype. Due to PET/CT, meanwhile, 54 patients (20.1%) were upstaged compared to BMB. Of these patients, 36 had DLBCL, 9 had follicular lymphoma, 7 had mantle cell lymphoma, 1 had PMBCL, and 1 had Burkitt’s lymphoma subtype.

### Sensitivity, Negative Predictive Value, and Accuracy of PET/CT and BMB

Both PET/CT and BMB were positive in 24 patients. In 54 patients, BMB was negative while PET/CT was positive, and in 42 patients, PET/CT was negative while BMB was positive. The PET/CT sensitivity was 65% [95% confidence interval (CI): 55.76-73.48] and the NPV was 78.13% (95% CI: 73.67-82.01). The sensitivity and NPV of BMB were 55% (95% CI: 45.65-64.09) and 73.53% (95% CI: 69.51-77.2), respectively. PET/CT accuracy was 84.44% (95% CI: 79.56-88.55), whereas the accuracy of BMB was 80% (95% CI: 74.72-84.6). When the performance of the three measurements (sensitivity, NPV, and accuracy) in all patient subgroups was evaluated, PET/CT was found to be superior to BMB for the DLBCL and follicular lymphoma subtypes, whereas for the mantle cell lymphoma, Burkitt’s lymphoma, and PMBCL subtypes BMB was superior to PET/CT. The values of all measurements in the NHL subgroups are shown in Table 2. In this group of patients, a statistically significant concordance was seen between BMB and PET/CT with respect to BMI (kappa value: 0.125; p<0.001).

### Group II - Hodgkin’s Lymphoma

In this group, 110 patients were evaluated and the median age was 38 years (range: 19-79). Forty patients were female and 70 patients were male. While 13 (11.8%) of the patients had BMI in BMB, 21 (19.1%) patients had BMI according to PET/CT findings. Fourteen of 21 patients had focal involvement and 7 patients had multifocal involvement. In the 11 patients who had both positive BMB and PET/CT, there was focal involvement in 8 of them and multifocal involvement in 3 of them in PET/CT. Of 10 patients who were negative for BMB but positive for PET/CT, 8 had focal involvement and 2 had multifocal involvement.

### Impact of PET/CT and BMB Findings on Staging

According to PET/CT findings, 7 patients were evaluated as stage 1, 29 patients as stage 2, 52 patients as stage 3, and 23 patients as stage 4. Two patients, one with stage 2 and the other one with stage 3, were considered to be stage 4 because of positive BMB. With BMB, 2 (1.8%) patients were protected from downstaging.

### Sensitivity, Negative Predictive Value, and Accuracy of PET/CT and BMB

Both PET/CT and BMB were positive in 11 patients. BMB was negative in 10 patients while PET/CT was positive, and in 2 patients BMB was positive while PET/CT was negative. PET/CT sensitivity was 91.3% (95% CI: 71.96-98.93) and NPV was 97.75% (95% CI: 92.05-99.39). The sensitivity and negative predictive value of BMB were 56.52% (95% CI: 34.49-76.81) and 89.69% (95% CI: 84.52-93.27), respectively. PET/CT accuracy was 98.18% (95% CI: 93.59-99.78), while the accuracy of BMB was 90.91% (95% CI: 83.92-95.55). A comparison of BMB and PET/CT results in detecting BMI in the whole patient population is shown in Table 3. The concordance between BMB and PET/CT with respect to BMI in this group of patients was also found to be significant (kappa value: 0.215; p<0.001).

## Discussion

In our NHL and HL series of 379 patients, we evaluated the performance of PET/CT and BMB in detecting BMI at the time of diagnosis. Our results reveal that PET/CT has a high accuracy rate in showing BMI in HL patients and significantly eliminates the need for BMB in this group of patients. However, this does not apply to the NHL patient group. BMB, which plays an important role in lymphoma staging, is an invasive method based on the examination of a very small area. BMI ascertained by BMB is detected in approximately 3%-18% of HL cases [15], while it is observed in NHL at rates between 29% and 69% [16]. In our study, while BMI ascertained by BMB was found to be 24.5% in NHL patients, this rate was 11.8% in HL patients. PET/CT is a highly sensitive and specific imaging method for detecting extranodal involvement and monitoring response to treatment [17,18]. In our study, 28.9% of NHL patients and 19.1% of HL patients were found to have BMI via PET/CT. The PET/CT BMI rates that we identified were lower than those found by Özpolat et al. [19], who detected BMI by PET/CT in 50% of NHL patients and 62% of HL patients. Statistically significant concordance was found between PET/CT and BMB according to BMI in both patient groups. As in the study conducted by Çetin et al. [20], concordance in the HL patient group was stronger than in the NHL patient group. Looking at the whole patient group and subgroups of NHL, we find quite interesting results. First of all, 15.6% of patients with histologically proven BMI identified by BMB could not be confirmed by PET/CT. If the BMB had not been performed, there would have been a risk of downstaging and under-treatment. In the whole group of patients with NHL, although the performance of PET/CT in DLBCL and follicular lymphoma grade 3 subtypes was better than BMB in determining BMI, BMB was more successful for mantle cell lymphoma, Burkitt’s lymphoma, and PMBCL subtypes. In the study conducted by Chen-Liang et al. [21], which calculated performance criteria using a similar method, BMB was more successful in detecting BMI in the entire NHL patient group, in follicular lymphoma grade 3, and in mantle cell lymphoma compared to PET/CT, while for DLBCL and Burkitt’s lymphoma, PET/CT was found to be more successful. Similar to our results, in this study, there was over 60% sensitivity in detecting BMI using PET/CT in DLBCL, which is the largest subgroup of NHL. As suggested by the latest Lugano classification [22], it would be appropriate to remove routine BMB from staging in DLBCL cases when PET/CT is positive for BMI. On the other hand, if PET/CT is negative for BMI, BMB should be recommended to these patients, considering that 15.6% histologic discordance would affect patient management. These recommendations are valid for the follicular lymphoma grade 3 subgroup with similar results in our study. A combination of PET/CT and BMB will be appropriate for the staging of subtypes of mantle cell lymphoma, Burkitt’s lymphoma, and PMBCL with PET/CT sensitivity below 45% in BMI detection.

The sensitivity of PET/CT in detecting BMI in HL is considerably higher than that of BMB. Considering the small number of patients who will not receive optimal treatment due to downstaging, the need for BMB seems to have disappeared during routine HL staging, consistent with recent studies [23,24]. However, BMB should be recommended in patients with cytopenia and negative PET/CT.

### Study Limitations

The main limitation of our study was that our patient subgroups were not homogeneous and the number of patients for some subtypes was low. Regarding our PET/CT results, it may be noted that we cannot give maximum standardized uptake value (SUV_max_) measurements and that FDG avidity may be different in different subtypes, but inclusion of patients due to FDG avidity of 97%-100% for HL and DLBCL, 91%-100% for follicular lymphoma, and 100% for Burkitt’s lymphoma and mantle cell lymphoma, as well as exclusion of patients due to lower avidity of FDG in low-grade NHL and chronic lymphocytic leukemia/small lymphocytic lymphoma [25], supports our study design.

## Conclusion

Considering the test results, we recommend considering BMB in PET/CT-negative cases in DLBCL and follicular lymphoma grade 3 subtypes for BMI evaluation and co-administration of PET/CT and BMB in cases of mantle cell lymphoma, Burkitt’s lymphoma, and PMBCL. We think that PET/CT and BMB are still complementary tests for NHL in assessing BMI. In cases of HL, we recommend using PET/CT due to its high accuracy for BMI assessment and to avoid BMB.

## Figures and Tables

**Table 1:**
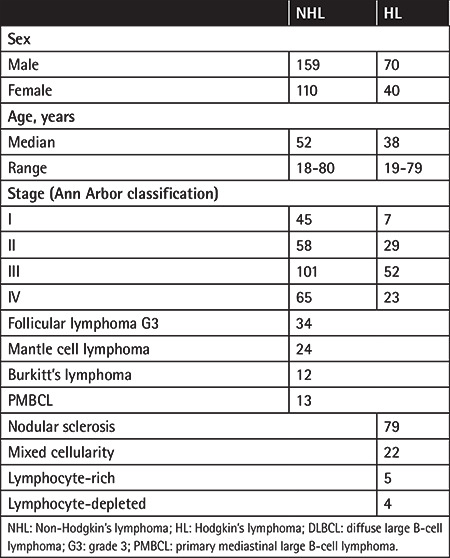
Baseline characteristics of patients included in the study.

**Table 2:**
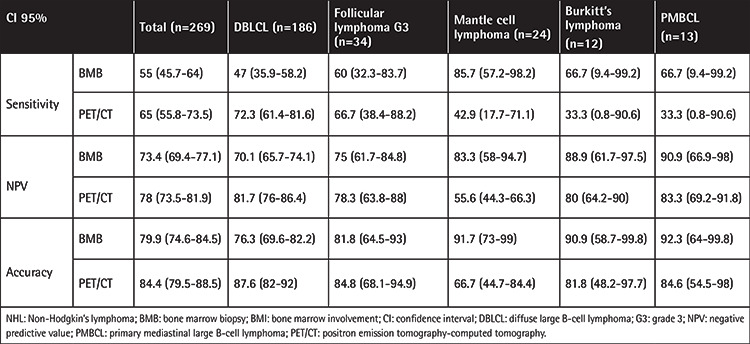
Performance of BMB and PET/CT in determining BMI in NHL patients.

**Table 3:**
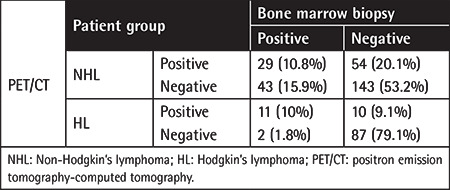
Detection of bone marrow involvement in lymphoma cases.

## References

[1] Hiddemann W, Dreyling M, Stahel RA;, ESMO Guidelines Task Force (2005). Minimum clinical recommendations for diagnosis, treatment and follow-up of newly diagnosed follicular lymphoma. Ann Oncol.

[2] Jost LM, Stahel RA;, ESMO Guidelines Task Force (2005). ESMO minimum clinical Recommendations for diagnosis, treatment and follow-up of Hodgkin’s disease. Ann Oncol.

[3] Zhang QY, Foucar K (2009). Bone marrow involvement by Hodgkin and non-Hodgkin lymphomas. Hematol Oncol Clin North Am.

[4] Brunetti GA, Tendas A, Meloni E, Mancini D, Maggiore P, Scaramucci L, Giovannini M, Niscola P, Cartoni C, Alimena G (2011). Pain and anxiety associated with bone marrow aspiration and biopsy: a prospective study on 152 Italian patients with hematological malignancies. Ann Hematol.

[5] Bain BJ (2005). Bone marrow biopsy morbidity: review of 2003. J Clin Pathol.

[6] Kim HY, Kim JS, Choi DR, Kim HS, Kwon JH, Jang GD, Kim JH, Jung JY, Song HH, Lee YK, Min SK, Hwang HS, Kim HJ, Zang DY, Kim HJ (2015). The clinical utility of FDG PET-CT in evaluation of bone marrow involvement by lymphoma. Cancer Res Treat.

[7] Chen YK, Yeh CL, Tsui CC, Liang JA, Chen JH, Kao CH (2011). F-18 FDG PET for evaluation of bone marrow involvement in non-Hodgkin lymphoma: a meta-analysis. Clin Nucl Med.

[8] Koh Y, Lee JM, Woo GU, Paeng JC, Youk J, Yoon SS, Kim I, Kang KW (2019). FDG PET for evaluation of bone marrow status in T-cell lymphoma. Clin Nucl Med.

[9] Pelosi E, Penna D, Douroukas A, Bellò M, Amati A, Arena V, Passera R, Bisi G (2011). Bone marrow disease detection with FDG-PET/CT and bone marrow biopsy during the staging of malignant lymphoma: results from a large multicentre study. Q J Nucl Med Mol Imaging.

[10] Angelopoulou MK, Mosa E, Pangalis GA, Rondogianni P, Chatziioannou S, Prassopoulos V, Moschogianni M, Tsirkinidis P, Asimakopoulos JV, Konstantinou I, Tsourouflis G, Sachanas S, Yiakoumis X, Boutsikas G, Arapaki M, Gainaru G, Kyrtsonis MC, Siakantaris MP, Datseris I, Panayiotidis P, Konstantopoulos K, Vassilakopoulos TP (2017). The significance of PET/CT in the initial staging of Hodgkin lymphoma: experience outside clinical trials. Anticancer Res.

[11] Teagle AR, Barton H, Charles-Edwards E, Dizdarevic S, Chevassut T (2017). Use of FDG PET/CT in identification of bone marrow involvement in diffuse large B cell lymphoma and follicular lymphoma: comparison with iliac crest bone marrow biopsy. Acta Radiol.

[12] Zhou M, Chen Y, Liu J, Huang G (2018;19). A predicting model of bone marrow malignant infiltration in 18F-FDG PET/CT images with increased diffuse bone marrow FDG uptake. J Cancer.

[13] Asenbaum U, Nolz R, Karanikas G, Furtner J, Woitek R, Simonitsch-Klupp I, Raderer M, Mayerhoefer ME (2018). Bone marrow involvement in malignant lymphoma: evaluation of quantitative PET and MRI biomarkers. Acad Radiol.

[14] Khan AB, Barrington SF, Mikhaeel NG, Hunt AA, Cameron L, Morris T, Carr R (2013). PET-CT staging of DLBCL accurately identifies and provides new insight into the clinical significance of bone marrow involvement. Blood.

[15] Sudalaimuthu M, Basu D (2017). Clinicopathological features of bone marrow infiltration in Hodgkin lymphoma. Should bone marrow staging be done only in high risk patients?. Turk Patoloji Derg.

[16] Lim EJ, Peh SC (2000). Bone marrow and peripheral blood changes in non-Hodgkin’s lymphoma. Singapore Med J.

[17] Freudenberg LS, Antoch G, Schütt P, Beyer T, Jentzen W, Müller SP, Görges R, Nowrousian MR, Bockisch A, Debatin JF (2004). FDG-PET/CT in re-staging of patients with lymphoma. Eur J Nucl Med Mol Imaging.

[18] Fuertes S, Setoain X, López-Guillermo A, Montserrat E, Fuster D, Paredes P, Lomeña F, Pons F (2007). The value of positron emission tomography/computed tomography (PET/CT) in the staging of diffuse large B-cell lymphoma. Med Clin (Barc).

[19] Özpolat HT, Yilmaz E, Goksoy HS, Özpolat S, Dogan Ö, Unal SN, Nalcaci M (2018). Detection of bone marrow involvement with FDG PET/CT in patients with newly diagnosed lymphoma. Blood Res.

[20] Çetin G, Çıkrıkçıoğlu MA, Özkan T, Karatoprak C, Ar MC, Eşkazan AE, Ayer M, Cerit A, Gözübenli K, Uysal BB, Erdem S, Ergül N, Tatar G, Çermik TF (2015). Can positron emission tomography and computed tomography be a substitute for bone marrow biopsy in detection of bone marrow involvement in patients with Hodgkin’s or non-Hodgkin’s lymphoma?. Turk J Hematol.

[21] Chen-Liang TH, Martin-Santos T, Jerez A, Senent L, Orero MT, Remigia MJ, Muiña B, Romera M, Fernandez-Muñoz H, Raya JM, Fernandez-Gonzalez M, Lancharro A, Villegas C, Carlos Herrera J, Frutos L, Luis Navarro J, Uña J, Igua C, Sanchez-Vaño R, Cozar Mdel P, Contreras J, Sanchez-Blanco JJ, Perez-Ceballos E, Ortuño FJ (2015). The role of bone marrow biopsy and FDG-PET/CT in identifying bone marrow infiltration in the initial diagnosis of high grade non-Hodgkin B-cell lymphoma and Hodgkin lymphoma. Accuracy in a multicenter series of 372 patients. Am J Hematol.

[22] Cheson BD, Fisher RI, Barrington SF, Cavalli F, Schwartz LH, Zucca E, Lister TA (2014). Recommendations for initial evaluation, staging, and response assessment of Hodgkin and non-Hodgkin lymphoma: the Lugano classification. J Clin Oncol.

[23] Cerci JJ, Bogoni M, Buccheri V, Etchebehere ECSC, Silveira TMBD, Baiocchi O, Neto CACP, Sapienza MT, Marin JFG, Meneghetti JC, Novis Y, Souza CA, Chiattone C, Torresan M, Ramos CD (2018). Fluorodeoxyglucose-positron emission tomography staging can replace bone marrow biopsy in Hodgkin’s lymphoma. Results from Brazilian Hodgkin’s Lymphoma Study Group. Hematol Transfus Cell Ther.

[24] Richardson SE, Sudak J, Warbey V, Ramsay A, McNamara CJ (2012). Routine bone marrow biopsy is not necessary in the staging of patients with classical Hodgkin lymphoma in the 18F-fluoro-2-deoxyglucose positron emission tomography era. Leuk Lymphoma.

[25] Barrington SF, Mikhaeel NG, Kostakoglu L, Meignan M, Hutchings M, Müeller SP, Schwartz LH, Zucca E, Fisher RI, Trotman J, Hoekstra OS, Hicks RJ, O’Doherty MJ, Hustinx R, Biggi A, Cheson BD (2014). Role of imaging in the staging and response assessment of lymphoma: Consensus of the international conference on malignant lymphomas imaging working group. J Clin Oncol.

